# Maturity in enterprise data warehouses for research operations: Analysis of a pilot study

**DOI:** 10.1017/cts.2023.23

**Published:** 2023-02-20

**Authors:** Boyd M. Knosp, David A. Dorr, Thomas R. Campion

**Affiliations:** 1 Roy J. and Lucille A. Carver College of Medicine and the Institute for Clinical & Translational Science, University of Iowa, Iowa City, IA, USA; 2 Department of Medical Informatics and Clinical Epidemiology, Oregon Health & Science University, Portland, OR, USA; 3 Department of Medicine, Oregon Health & Science University, Portland, OR, USA; 4 Clinical & Translational Science Center, Weill Cornell Medicine, New York, NY, USA

**Keywords:** Maturity models, enterprise data warehouses for research, translational research, infrastructure, biomedical informatics, CTSA

## Abstract

Enterprise data warehouses for research (EDW4R) is a critical component of National Institutes of Health Clinical and Translational Science Award (CTSA) hubs. EDW4R operations have unique needs that require specialized skills and collaborations across multiple domains which limit the ability to apply existing models of information technology (IT) performance. Because of this uniqueness, we developed a new EDW4R maturity model based on prior qualitative study of operational practices for supporting EDW4Rs at CTSA hubs. In a pilot study, respondents from fifteen CTSA hubs completed the novel EDW4R maturity index survey by rating 33 maturity statements across 6 categories using a 5-point Likert scale. Of the six categories, respondents rated workforce as most mature (4.17 [3.67–4.42]) and relationship with enterprise IT as the least mature (3.00 [2.80–3.80]). Our pilot of a novel maturity index shows a baseline quantitative measure of EDW4R functions across fifteen CTSA hubs. The maturity index may be useful to faculty and staff currently leading an EDW4R by creating opportunities to explore the index in local context and comparison to other institutions.

## Introduction

Delivering patient data, such as that stored in electronic health record (EHR) systems, to scientists in a timely, secure, and useable manner is a crucial component of clinical and translational research; these data help investigators form new hypotheses, perform observational and real-world studies, and facilitate clinical trials. Providing these data has been one of the core functions of informatics teams at the National Institutes of Health Clinical and Translational Science Award (CTSA) hubs. The Enterprise Data Warehouse for Research (EDW4R) [1,2] is the ecosystem that hubs use to deliver this data.

**Fig. 1.**
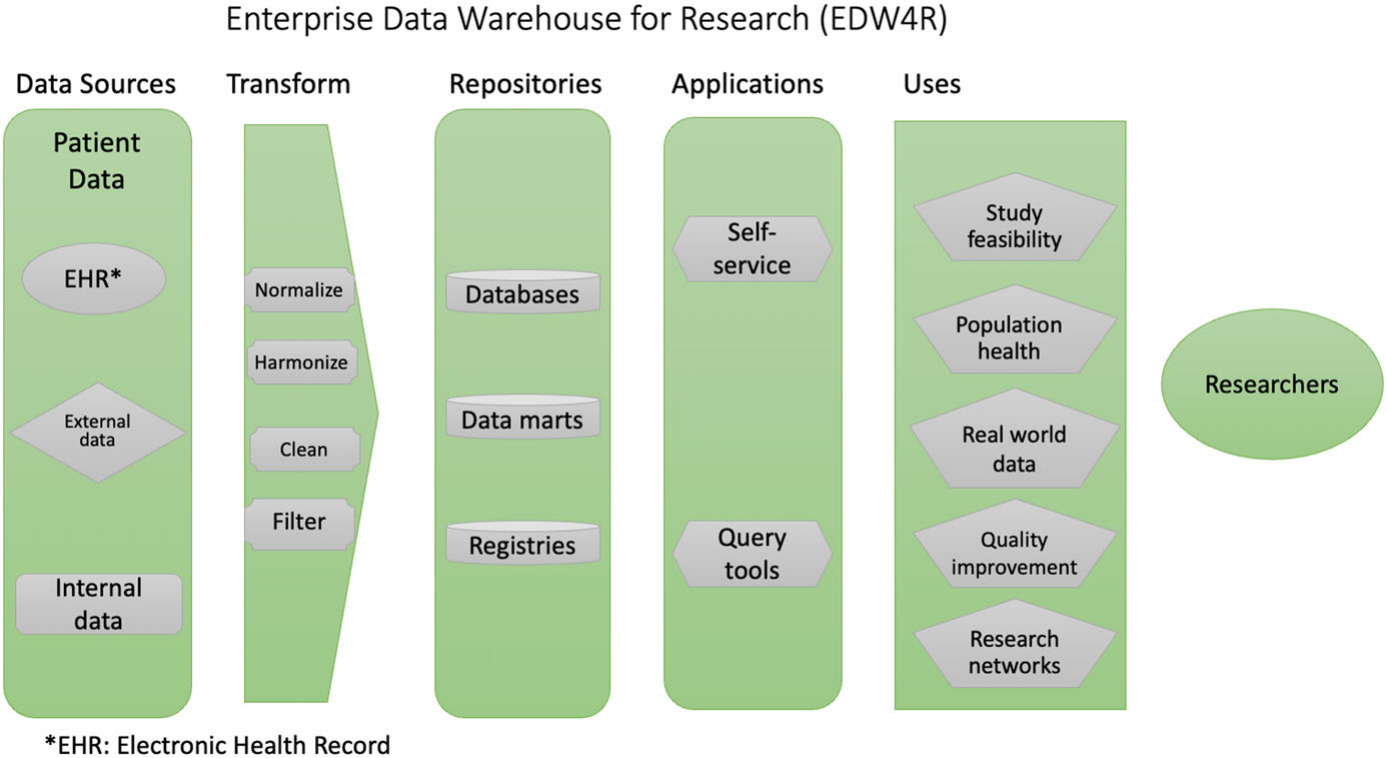
Enterprise Data Warehouse for Research (EDW4R) framework.

As shown in Fig. 1, EDW4Rs are the technologies and processes CTSA hubs use to deliver patient data for analysis to clinical and translational researchers. They aggregate data from EHR systems as well as other sources including external (e.g., insurance claims, social determinants of health) and internal (e.g., genomics, biospecimens, patient-reported outcomes) data sets. These data are then transformed into a format that is accessible for research and is stored in some protected data repository, which is often a data warehouse, a structured collection of historical data designed for analytics, but may be a data lake, a raw collection of unaggregated data, or a copy of the transactional database that is set aside for research use. These repositories are then queried – often by data team staff or by investigators using self-service tools – and the resulting extracts are used in a number of clinical and translational science uses including but not limited to study feasibility, population health, real-world evidence generation, and sharing in research networks.

In recent years, EDW4R has supported a wider spectrum of research uses going beyond clinical trial recruitment [3] to enable efforts that develop, validate, and disseminate new and increasingly complex algorithms, perform observational studies (e.g., comparative effectiveness meta-studies or phenome-wide association studies [4]), real-world data analyses (e.g., pharmacovigilance [5]), and pragmatic trials [6]. EDW4R has also been used to drive multi-institutional data studies through research networks such as PCORnet [7,8], ACT [9], and OHDSI [10] and to contribute to national-level aggregation of data for specific emergent health crisis [11].

Our previous work has shown that EDW4R operations vary across the CTSA consortium, and optimal approaches to EDW4R are unknown [1,2]. This has made it challenging for CTSA hubs to deliver efficient and effective service and support and adapt to ever-changing requirements. To address this challenge, some form of modeling of EDW4R operations is needed to guide the development and tracking of institutional EDW4R processes and organization and compare them to practices at other institutions.

Maturity models [12–14] are systematic methods to explain and track how organizations and processes develop. They have been used to provide guidance for a wide spectrum of information technology (IT) and in particular have been used to guide research IT and informatics development [15], clinical analytics [16], and EHR system adoption [17]. In academic medical centers, Chief Information Officers (CIOs) may be familiar with the Healthcare Information and Management Systems Society (HIMSS) Electronic Medical Record Adoption Model, which has been used by thousands of healthcare organizations to assess and guide digital maturity with respect to implementation of and investment of clinical information systems [17].

A maturity index [18] measures organizational capacity to deliver a service, considering multiple factors including culture, policy, and organization. The indices are generally described in order of increasing maturity, with similar level descriptors across models. For instance, a 5-level model is common, with level 1 described as “initial” or “ad hoc” (i.e., unpredictable and poorly controlled processes) and level 5 as “optimizing” (i.e., continual improvement of well-managed processes). In university settings, CIOs may be familiar with the maturity index used by EDUCAUSE (https://educause.edu), a 2000+ member non-profit organization dedicated to advancing higher education through information technology. EDUCAUSE’s Core Data Service [18] benchmarks organizational IT practices including organizational maturity. Hundreds of academic institutions have used this resource [18]. The HIMSS and EDUCAUSE maturity measurement experiences suggest that EDW4R operations could benefit from the development of an EDW4R maturity model.

EDW4R operations have unique needs that require specialized skills and collaborations across multiple domains – scientific, clinical, information technology, and research compliance among others – which limit the ability to apply existing models of IT and clinical data performance. To address this gap, we developed and piloted a new EDW4R maturity model.

## Methods

### Instrument Development

We developed a maturity index that is based on the “hybrids and Likert-like questionnaires” maturity model group as described by Fraser [12] rather than a “capability maturity model” or a “maturity grid” approach. Each item in this index is a statement of best practice, and a respondent scores their organization’s relative performance toward achieving that practice on a five-point scale. The statements are grouped into *categories* that provide a multi-dimensional set of ratings. These best practice statements, called *maturity anchor statements*, aim to define the “Optimized” state for different characteristics identified across each category. This type of maturity index, which is used in the EDUCAUSE Core Data Service [18] upon which we modeled our index, allows two institutions to be at the same level of maturity but to have achieved it in different ways, which reflects EDW4R operational variation previously observed across the CTSA consortium [1,2].

Leveraging our prior analysis of 40 interviews with CTSA hubs [1,2] as well as discussions at more than 20 CTSA EDW4R working group (WG) meetings with informatics leaders, we developed a maturity index by identifying several categories that reflect topical activities for EDW4R operations. These categories were created initially from early discussions with EDW4R WG members and evolved as we completed two rounds of interviews and subsequent analysis of the interview summaries. The final categories were reviewed with the EDW4R WG and modified based on their feedback. The six categories were as follows: **access and outreach** (researcher engagement and access strategies); **service management** (level of formalization of services); **workforce** (staffing characteristics of EDW4R team); **relationship to enterprise IT** (engagement of EDW4R team with central IT units); **data governance (**decision-making about EDW4R); and **metrics** (measurement of EDW4R operations).

Within each *category*, we developed four to six *maturity anchor statements* [10], descriptions of best practices that are ratable using a Likert scale. We reviewed the statements with our EDW4R WG, addressing statement clarity and removing any ambiguous statements from the pool. The index we developed consisted of six *categories* with 33 *maturity anchor statements* total.

### Data Collection and Analysis

Using REDCap [19], we created a survey with a Likert scale rating for the 33 anchor statements (REDCap data dictionary of the index is available at https://bit.ly/EDW4R_Maturity). For each statement in the survey, “strongly agree” responses indicated the highest maturity rating (5) and “strongly disagree” indicated the lowest maturity rating (1) with “agree,” “neutral,” and “disagree” as options ranging from 4 to 2, respectively. All statements also had the “not applicable” response option. We invited informatics leads from CTSA hubs via email to complete the survey, which was available from January 1, 2021, through February 28, 2021. Within and across categories, we then determined descriptive statistics, including median (interquartile range (IQR)).

## Results

**Fig. 2.**
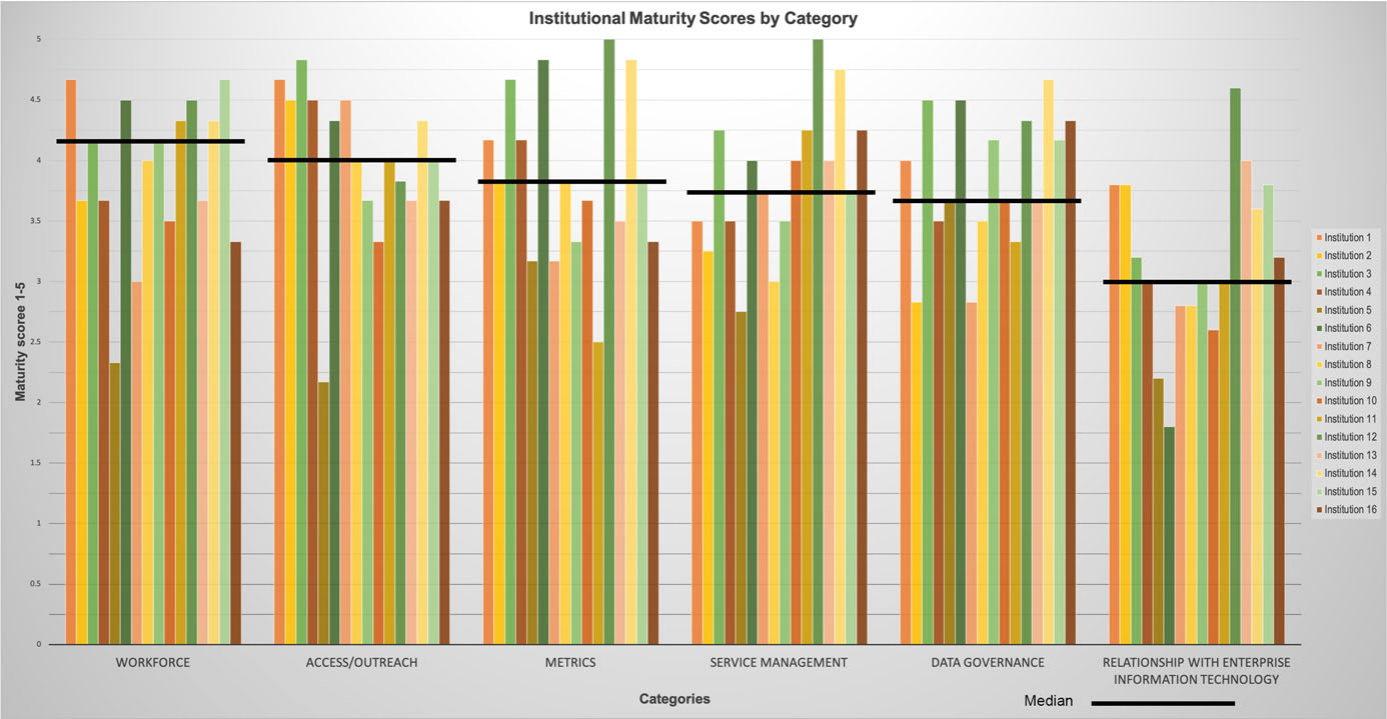
Maturity scores across all six categories colored by institution.

Fifteen CTSA hubs (more than 25% of the total CTSA hubs in the USA) completed the survey assessing maturity of EDW4R operations. Fig. 2 shows maturity scores for the six categories colorized by each institution. Notably, respondents rated **workforce** as most mature (4.17 [3.67–4.42]) followed by access and outreach (4.00 [3.75–4.50]), metrics (3.83 [3.42–4.42]), service management (3.75 [3.50–4.13]), and data governance (3.67 [3.50–4.25]); **relationship with enterprise IT** was the least mature (3.00 [2.80–3.80]). Overall maturity was 3.72 [3.55–4.09].

Table 1 shows ratings of maturity anchor statements within each category sorted from highest to lowest median score. Also indicated for each statement is the number of responses, which varied, as some institutions did not respond to certain statements or selected “not applicable” in their response.

**Table 1.** Enterprise Data Warehouse for Research (EDW4R) Maturity anchor statements grouped by category and sorted by median score with interquartile range (IQR)

Category	#	Maturity Anchor Statement	*n*	Median [IQR]
Workforce	1.1	We have one or more staff whose duties include processing requests for data from our EDW4R.	15	5 [4.5–5]
	1.2	We have one or more staff whose duties include aggregating and managing the data stored in our EDW4R.	15	5 [4–5]
	1.3	We have one or more staff whose duties include project management of activities related to our EDW4R team.	14	4.5 [4–5]
	1.4	We have one or more staff whose duties are shared with enterprise IT.	15	4 [3–4.5]
	1.5	Our EDW4R team includes faculty domain experts who assist with EDW4R services.	15	4 [2.5–4.5]
	1.6	We have identified training programs as pipelines to fill open positions on our EDW4R team.	14	3 [2.25–4]
Access and Outreach	2.1	We require Collaborative Institutional Training Initiative or other relevant research ethics training prior to accessing our EDW4R.	15	5 [4.5–5]
	2.2	We have self-service tools for exploring a de-identified portion of the EDW4R.	14	5 [4.25–5]
	2.3	We are able to generate reports from our Electronic Health Record for research requests.	15	5 [4–5]
	2.4	We have a variety of methods for enabling users to access data in our EDW4R, which take into consideration the level of data expertise of the researcher.	14	4 [3.25–5]
	2.5	We offer a service that establishes a population-specific data mart that is periodically updated.	15	4 [3–4.5]
	2.6	We have regular orientation courses in accessing data in the EDW4R.	14	3 [3–4]
Metrics	3.1	We keep track of the number of research requests we receive for patient data.	15	5 [5–5]
	3.2	We assess our EDW4R using National Center for Advancing Translational Science’s Common Metrics.	14	5 [4.25–5]
	3.3	We track metrics that are used for strategic planning.	14	4 [3–4.75]
	3.4	We track response times for research requests for patient data.	15	4 [3–4.5]
	3.5	We track the outcomes (publications, grants…) resulting from research requests for patient data.	15	4 [3–4]
	3.6	We provide data quality assessments for a research network such as the National Patient-Centered Clinical Research Network or Observational Health Data Sciences and Informatics.	15	4 [2.5–5]
Service Management	4.1	We have a standard format for submitting data requests.	15	5 [4–5]
	4.2	We have a written description of the services available to access our EDW4R.	15	4 [4–5]
	4.3	Our IT helpdesk knows to refer research requests for clinical data to our EDW4R team	15	4 [3–4]
	4.4	Our EDW4R services are listed as part of our enterprise IT service catalog.	13	3 [2–4]
Data Governance	5.1	We manage requests for access to our EDW4R with guidance from our Institutional Review Board.	15	5 [4–5]
	5.2	We have a team that engages with the Institutional Review Board, compliance, and legal to define policies regarding requests for using data from the EDW4R.	15	4 [4–5]
	5.3	We have a team that defines what data from source systems goes into the EDW4R	14	4 [4–5]
	5.4	We have a team that reviews and prioritizes data requests to the EDW4R.	15	4 [3.5–5]
	5.5	We have a high-level committee that reviews external agreements regarding accessing data from the EDW4R.	15	3 [2.5–4]
	5.6	Our governance structure considers both clinical and research data requests.	15	3 [2–4]
Relationship with Enterprise IT	6.1	Our EDW4R group works closely with the clinical data warehouse teams.	15	4 [4–4]
	6.2	Our Chief Research Information Officer collaborates closely with other Executive-suite leaders.	14	4 [3–4]
	6.3	The EDW4R is part of the overall Enterprise IT strategic planning process.	14	4 [2.25–4]
	6.4	EDW4R services are listed as part of the Enterprise IT service catalog.	13	3 [2–4]
	6.5	Our EDW4R data team is integrated into our Enterprise IT organization.	15	3 [2–4]

IT, Information Technology.

## Discussion

Fifteen CTSA hubs participated in a pilot study of a new maturity index addressing six categories of EDW4R operations. Findings show variation in EDW4R operational maturity with a median (IQR) of 3.72 [3.55–4.09], indicating a potential baseline for tracking and comparing EDW4R maturity over time. The category results showed that participating institutions are most mature in **workforce** and **access and outreach** and least mature in **relationship with enterprise IT**. These results provide guidance into areas where individual institutions and the CTSA community can potentially focus to improve EDW4R operations.

The scores of the individual maturity anchor statements indicate specific areas where the participant group has growth opportunities, even in categories where the group’s maturity score is high. For example, in Table 1, statement 1.6 (“We have identified training programs as pipelines to fill open positions on our EDW4R team”) has a score of 3 [2.54–4], which is low compared to other statements in the category. This indicates that the participating institutions have an opportunity to grow in their workforce maturity to identify better recruitment pathways for EDW4R staffing.

The EDW4R index is built on our prior qualitative work that helped illustrate the complexity of EDW4R operation. The current study extends prior work by providing a method to quantify EDW4R complexity to support assessment across organizations and management of individual institutions. The maturity method we used, category-based maturity anchor statements, appears to appropriately address the current state of variable EDW4R development at CTSA hubs.

In non-healthcare sectors of the economy, data warehouse maturity has been well documented by Sen [20] who described a capability maturity model for data warehouse development and services that assume a typical enterprise level of investment in data warehouse operations. Our observations are that EDW4R development and services at CTSA hubs are an evolving set of processes driven by the needs of clinical and translational researchers and correlation between EDW4R operations and the data warehousing process maturity described by Sen is limited. There are lessons for CTSA hubs to learn from Sen’s model such as the separation of data warehouse development maturity from operations maturity and the focus on maturing processes. Currently, Sen’s model does not address many of the clinical and translational science aspects specific to EDW4R operations, such as biomedical research expertise involved; scientific outcomes; multi-departmental organizational collaborations needed for successful EDW4R operations (e.g., relationship with enterprise IT); and variations in organizational culture of academic medical centers.

There are limitations to the current investigation. Our study with the maturity index is a pilot, and further validation is required to establish the index as a standard tool for measuring EDW4R operations. The index is a proposed set of best practices, but it does not directly outline a path of development – which practices to implement first, which can wait until later. While the maturity index reflects our prior qualitative work, an optimal maturity index provides a roadmap for developing whatever resource is being assessed. All maturity index scores in this pilot were self-reported and many of the anchor statements used are subjective, which might encourage bias toward higher scores to maintain institutional reputation. In future applications of this index, normalization across the sample group could be done by selected external reviewers to reduce this bias.

This work is a logical next step for previous work [15] done by one of the authors (BK) and collaborators that proposed the use of maturity models to assess research information technology and informatics in academic medicine. The current EDW4R study describes an assessment that builds from the value proposition described in the prior research informatics maturity work, including identification of gaps in aligning clinical needs with research, optimizing cross-organizational research environments, developing guidelines to participate in emerging communities of practice, and enabling strategic review of local expertise and infrastructure capabilities.

Next steps for this work are to operationalize the index as a practice within the CTSA community, making it a yearly or every-other-year activity. Validating the index through reviewing results with participating institutions and engaging subject matter experts to review the results are also important next steps.

Our pilot of a novel maturity index shows a baseline quantitative measure of EDW4R functions across 15 CTSA hubs. The maturity index may be useful to faculty and staff currently leading an EDW4R by creating opportunities to explore the index in local context and comparison to other institutions.
